# Supplementary material for: Resolving the full spectrum of human genome variation using Linked-Reads
Source: Genome Res. 2019 Apr;29(4):635–45. doi: 10.1101/gr.234443.118 (PMC6442396; doi:10.1101/gr.234443.118)
Supplement: Supplemental Material [file supp_gr.234443.118_Supplemental_longranger-2.2.zip › longranger-2.2.tar.bz2!/lr2.2-public/lib/stan/cmdstan/stan/doc/api/html/dir_adda0d2b1be4bc1eb47d3b2cf56797af.html]

Stan: src/stan/prob/distributions/univariate/continuous Directory Reference


|  |  |
| --- | --- |
|  | Stan  2.5.0  probability, sampling & optimization |


- Main Page
- Related Pages
- Namespaces
- Classes
- Files

All Classes Namespaces Files Functions Variables Typedefs Enumerations Enumerator Friends Macros Pages

- src
- stan
- prob
- distributions
- univariate
- continuous

continuous Directory Reference

|  |  |
| --- | --- |
| Files | |
| file | beta.hpp [code] |
|  | |
| file | cauchy.hpp [code] |
|  | |
| file | chi\_square.hpp [code] |
|  | |
| file | double\_exponential.hpp [code] |
|  | |
| file | exp\_mod\_normal.hpp [code] |
|  | |
| file | exponential.hpp [code] |
|  | |
| file | frechet.hpp [code] |
|  | |
| file | gamma.hpp [code] |
|  | |
| file | gumbel.hpp [code] |
|  | |
| file | inv\_chi\_square.hpp [code] |
|  | |
| file | inv\_gamma.hpp [code] |
|  | |
| file | logistic.hpp [code] |
|  | |
| file | lognormal.hpp [code] |
|  | |
| file | normal.hpp [code] |
|  | |
| file | pareto.hpp [code] |
|  | |
| file | pareto\_type\_2.hpp [code] |
|  | |
| file | rayleigh.hpp [code] |
|  | |
| file | scaled\_inv\_chi\_square.hpp [code] |
|  | |
| file | skew\_normal.hpp [code] |
|  | |
| file | student\_t.hpp [code] |
|  | |
| file | uniform.hpp [code] |
|  | |
| file | von\_mises.hpp [code] |
|  | |
| file | weibull.hpp [code] |
|  | |


---

[ Stan Home Page ]

*© 2011–2014,
Stan Development Team.*
